# Tumor immune escape: extracellular vesicles roles and therapeutics application

**DOI:** 10.1186/s12964-023-01370-3

**Published:** 2024-01-02

**Authors:** Mahdi Ahmadi, Reza Abbasi, Jafar Rezaie

**Affiliations:** 1 Stem Cell Research Center, Tabriz University of Medical Sciences, Tabriz, Iran; 2 Department of Biology, Urmia University, Urmia, Iran; 3 Solid Tumor Research Center, Cellular and Molecular Medicine Institute, Urmia University of Medical Sciences, Urmia, Iran

**Keywords:** Immune escape, Immunotherapies, Extracellular vesicles, PDL-1, Engineered EVs

## Abstract

**Background:**

Immune escape, a process by which tumor cells evade immune surveillance, remains a challenge for cancer therapy. Tumor cells produce extracellular vesicles (EVs) that participate in immune escape by transferring bioactive molecules between cells.

**The main body of the abstract:**

EVs refer to heterogeneous vesicles that participate in intercellular communication. EVs from tumor cells usually carry tumor antigens and have been considered a source of tumor antigens to induce anti-tumor immunity. However, evidence also suggests that these EVs can accelerate immune escape by carrying heat shock proteins (HSPs), programmed death-ligand 1 (PD-L1), etc. to immune cells, suppressing function and exhausting the immune cells pool. EVs are progressively being evaluated for therapeutic implementation in cancer therapies. EVs-based immunotherapies involve inhibiting EVs generation, using natural EVs, and harnessing engineering EVs. All approaches are associated with advantages and disadvantages. The EVs heterogeneity and diverse physicochemical properties are the main challenges to their clinical applications.

**Short conclusion:**

Although EVs are criminal; they can be useful for overcoming immune escape. This review discusses the latest knowledge on EVs population and sheds light on the function of tumor-derived EVs in immune escape. It also describes EVs-based immunotherapies with a focus on engineered EVs, followed by challenges that hinder the clinical translation of EVs that are essential to be addressed in future investigations.

Video Abstract

**Supplementary Information:**

The online version contains supplementary material available at 10.1186/s12964-023-01370-3.

## Background

The term “Immune escape” or antigen escape refers to a process by which tumor cells evade immune cells' recognition and responses, therefore getting survival and developing into metastatic tumor [1]. The process of Immune escape involves the expression of ligands on tumors cells and the release of immunosuppression factors that block function and exhaust the immune cells pool [1]. The immunosuppressive microenvironment of a tumor has an imperative role in cancer development and even immunotherapy responses [2]. Since immune escape is a main factor for tumor growth, such immune checkpoint-associated proteins as programmed death-ligand 1 (PD-L1) and programmed death-1 (PD-1); and other molecules have become the topic of extreme examination [3, 4]. Cancer is a large group of diseases that influences human society and the healthcare system [5, 6]. Recent progress in tumor cell biology has revealed the key functions of extracellular vesicles (EVs) in regulating immune responses and the immune escape of cancer cells [7]. EVs are double-phospholipid vesicles released by various tumor cells participate in cell communication [8, 9]. They contain multiple ranges of biomolecules on the surface of their lumen, carrying between cells, and exchanging information [8, 9]. The term EVs is wide-ranging and can encompass numerous vesicles like exosomes, ectosomes, and other different types of vesicles that are released by various cells [10]. In this regard, the International Society for Extracellular Vesicles (ISEV) was established in 2011, which sponsored the improvement and application of different EVs. In 2014 and then in 2018, the paper ‘Minimal Information for Studies of EVs’ (MISEV) guidelines was released for the standardization of this field regarding terms, isolation methods, characterization methods, and applications in preclinical and clinical trials [11, 12]. Tumor-derived EVs function as a double-edged sword since they can promote cancer growth and metastasis by lessening cytotoxicity, causing remodeling, and conserving immunosuppressive tumor microenvironment as well as can make up anti-cancer immune responses by delivering tumor antigens and various heat shock proteins (HSPs) like HSP90 and HSP70 [13, 14]. PD-L1 has been reported on tumor derived EVs, which may act like those of cancer cells, inducing immune escape [15]. Although the immunosuppressive impact of EVs-PD-L1 is confirmed; however, EVs-PD-L1 have positive effects. For example, the inhibitory role of PD-L1 could support wound healing and tissue repair [16]. Because acute pro-inflammatory conditions after trauma may worsen tissue harm [17]. There are still several problems that need to be considered in cancer therapy, like the escapes of immune surveillance and immune cell suppression [18, 19]. In recent years, to overcome immune escape, EVs-based therapies have emerged, for example inhibiting EVs generation by tumor cells or using immune cells-derived natural EVs approaches [20, 21]. Along with the advance and success of EVs-based research, engineered EVs are appealing to growing attention, particularly in tumor cell escape, because of their loading and temporal targeting aptitude [22] (Table 1). Each of these methods is associated with advantages and disadvantages (Table 2). For clinical translation, many steps are needed because EVs are heterogeneous in size, function and physicochemical properties [10]. Besides, the process engineering requires optimal methods regarding the type of EVs and loading methods as well as the type of cargo (see reviews [23–25]). This review aims to weigh the potential of EVs in inducing immune escape and highlights the significance of EVs experiments for beneficial applications in immune escape. First, we define EVs biology and heterogeneity. Next, the function of tumor derived EVs that cause immune escape will be discussed. Further sections will describe possible application of EVs for immune escape, natural EVs and engineered EVs, highlighting challenges for promising clinical application.

**Table 1:** Facts of extracellular vesicles and their role in immune escape mechanism and treatment

**Facts**
• Extracellular vesicles (EVs) are heterogeneous in route of generation, size, function, and cargo
• EVs participate in several physiological and pathological conditions
• tumor-derived extracellular vesicle (T-EVs) can contribute to immune escape directly or indirectly
• T-EVs contain PD-L1 and other biomolecules that suppress the function of immune cells and induce cell death in immune cells, inducing immune escape
• EVs can be harnessed to overcome immune escape, for example, EVs-based therapies, prevention of EVs biogenesis, and using engineered EVs
• For EVs-based therapies, T-EVs and EVs of DCs can be used as cancer vaccines, which stimulate immune responses
• For the prevention of EVs biogenesis, different agents may inhibit the biogenesis and uptake of EVs from cancerous cells
• For using engineered EVs, EVs from different sources can be modified or loaded with therapeutic agents inducing immune responses and proliferation
• Engineered EVs show promising results because they efficiently accumulate in tumor sites and profoundly stimulate immune cells

**Table 2:** Challenge in extracellular vesicles-based studies

**Gaps**
• Extracellular vesicles (EVs) are heterogeneous, therefore nomenclature, classification, isolation, and characterization of them remain a challenge
• Although tumor-derived extracellular vesicles (T-EVs) induce immune escape, however, there is evidence that T-EVs promote immune responses because they carry tumor antigens. This may arise from the type of EVs or tumor cells
• For EVs-based therapies, firstly, EVs must be produced in a large-scale manner and purified for downstream experiments accurately
• Although EVs from immune cells or tumor cells can induce immune responses, however, the risk of tumorigenesis remains a challenge. In addition, although the cancer vaccine was investigated in patients, the efficacy of these EVs is not satisfactory and dependent on cancer grade
• For the prevention of EVs biogenesis, different agents may inhibit the formation and secretion of certain EVs, however, these agents may cause side effects on the body and block healthy EVs. Thus, selecting a certain agent that inhibits EVs generation even uptake only from tumor cells remains a gold standard for this purpose. In addition, tumor cells release different types of EVs, thereby an agent may inhibit a type of EVs such as exosomes or microvesicles
• For using engineered EVs, many loading and engineering methods have been used by several laboratories; thereby there is a need for an optimized method
• The engineering methods may harm EVs structure, bio-distribution, and even function. Thus, further studies need to address these limitations
• Which EVs are suitable for drug delivery and engineering- is a main question in this field. In addition, the side effects and unwanted results may be associated with engineered EVs

### Extracellular vesicles

**Fig. 1:**
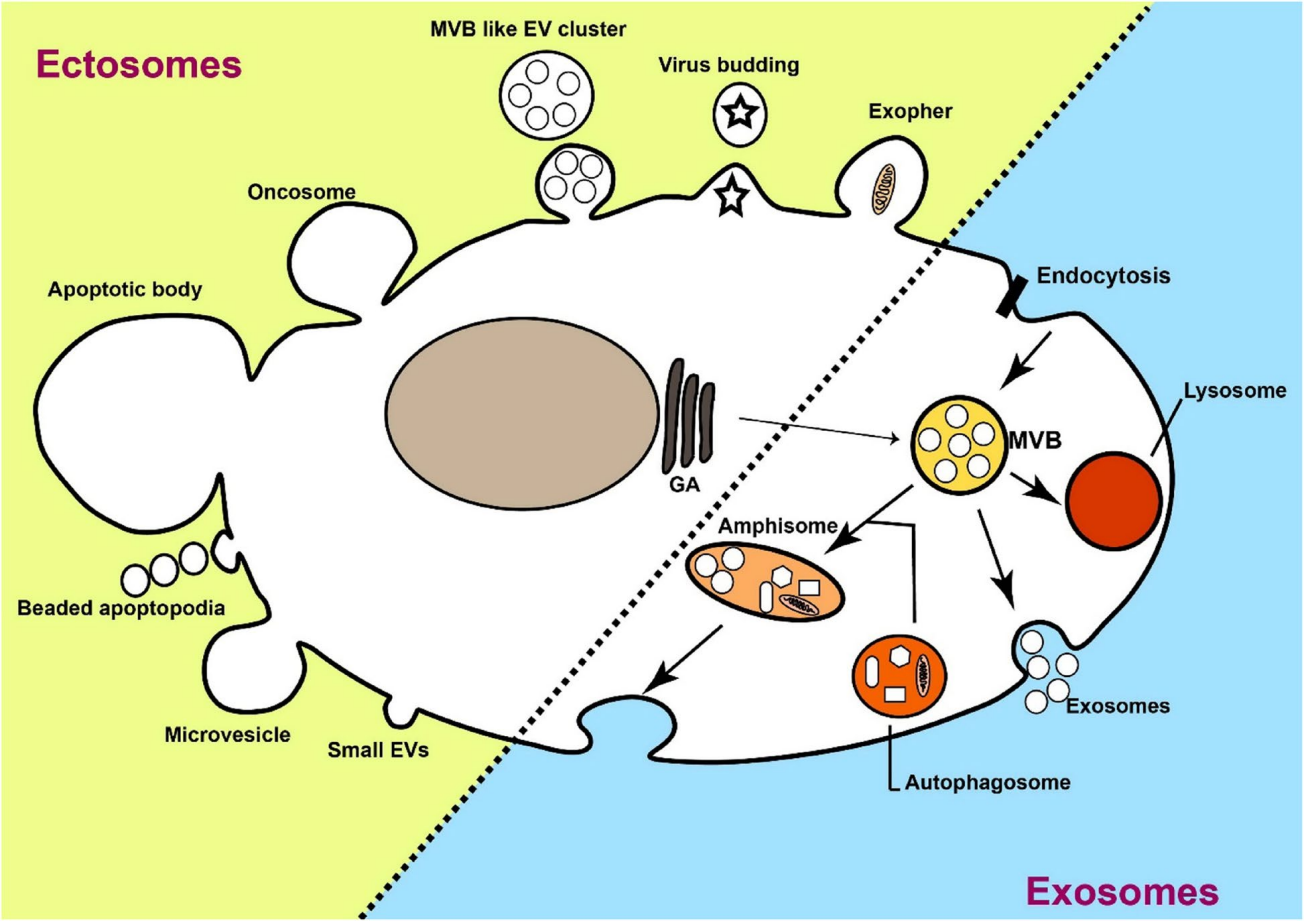
Heterogeneity in extracellular vesicles (EVs). Cells produce various types of EVs, which are different in route of biogenesis, shape, size, and cargo. In general, EVs may divided into two major groups including ectosomes and exosomes; both contain subgroups. Exosomes are the most common EVs that considerably were investigated in cell culture and animal models. Multivesicular bodies (MVBs) within cells are a place where exosomes are generated and loaded with biological cargo. Rather than a secretory pathway, MVBs may fuse with lysosomes or autophagasomes vesicles to produce amphisomes. Exosomes have subpopulations themselves based on size and cargo. Other EVs such as microvesicles, apoptotic bodies and other small EVs are generated by cells. Similar to exosomes, microvesicles have been studied for their pivotal roles in immune responses and drug delivery systems

Many eukaryotic cells communicate with other cells through the interchange of EVs [26]. EVs, a population of heterogeneous vesicles, contain phospholipid bilayer-membrane encircled different types of biomolecules that can be captured by recipient cells located either adjacent or distant [26]. However, it remains uncertain whether cells release EVs principally to evacuate cellular waste or unnecessary products, for intercellular communication, for cargo delivery, for spreading disease, or a combination of all [27–29]. Because EVs pathway shows crosstalk with other cellular signaling pathways [30]. However, EVs are broadly considered the most important factor in regulating physiological and pathological milieu. A growing body of literature has shown that EVs can affect target cells function in several ways including, internalization pathways, cargo delivery into the cytoplasm by direct fusion, and ligand-receptor interactions [31, 32]. The term EVs is general and comprise heterogeneous subpopulations of cell-derived particles with various size and morphologies [10]. The most famous subpopulations of EVs include exosomes and ectosomes [33, 34]. Exosomes can be divided into subpopulations; however, their range size is 30 to 150 nm, originating from endocytosis pathways within multivesicular bodies (MVBs) where several complexes and molecules are participating in forming intraluminal vesicles (ILVs) and loading biological cargo into ILVs. When ILVs within MVBs are released out of cell so-called exosomes, which process needs fusion of MVBs with the plasma membrane [35]. Not all ILVs culminate in to be exosomes, although ILVs are originators of exosomes. Alternatively, MVBs may fuse with lysosomes for degradation ILVs, even with autoghosomes [36]. A hybrid of exosomes and autophagosome form amphisomes, which also can release exosomes [37]. A growing body of evidence suggests that different MVB populations are present within a cell, proposing ILV subpopulations for degradation or elimination, then the regulation of this balance is not clear [8, 38, 39]. Interestingly, when degradation by lysosomes was inhibited, exosome production was increased, representing that these MVBs also have abilities to release ILVs as exosomes [40, 41]. Various ILV generation- and loading mechanisms have been suggested, which result in subpopulations of MVBs/exosomes. Besides, it was suggested that a single MVBs may contain different ILVs subpopulations [42]. It seems that exosome cargo loading is a regulated process and various mechanisms participate. Such markers as LAMP1/2, syntenin, various proteins from the ESCRT complex, CD81, CD9, and CD63 are often reported as specific markers for exosomes [43, 44]. Another EVs family is ectosomes, for example, microvesicles; which originate by blebbing of the plasma membrane, showing the composition of the plasma membrane [45]. Various ectosome subpopulations, produced via diverse biogenesis pathways, have been defined during several physiological cell stages or by many cell types [34] (Fig. 1). Ectosomes contain cell membrane markers and are very heterogeneous in size. Some typical markers such as SLC3A2, ARF6, annexin A1/2, and basigin, as well as CD9 and CD81, have been suggested to be the most specific [43]. Overall, because of the heterogeneous nature of EVs, they play a multipurpose function in physiological and pathological conditions [46–48]. EVs contribute to regulating different types of diseases such as cancers. EVs from Immune cells are also heterogeneous in route of biogenesis, size and cargo and are present in the blood, saliva, cerebrospinal fluid, and urine [49, 50], participating in different dimensions of tumors.

### Role of EVs in immune escape

#### EVs carrying PD-L1

**Fig. 2:**
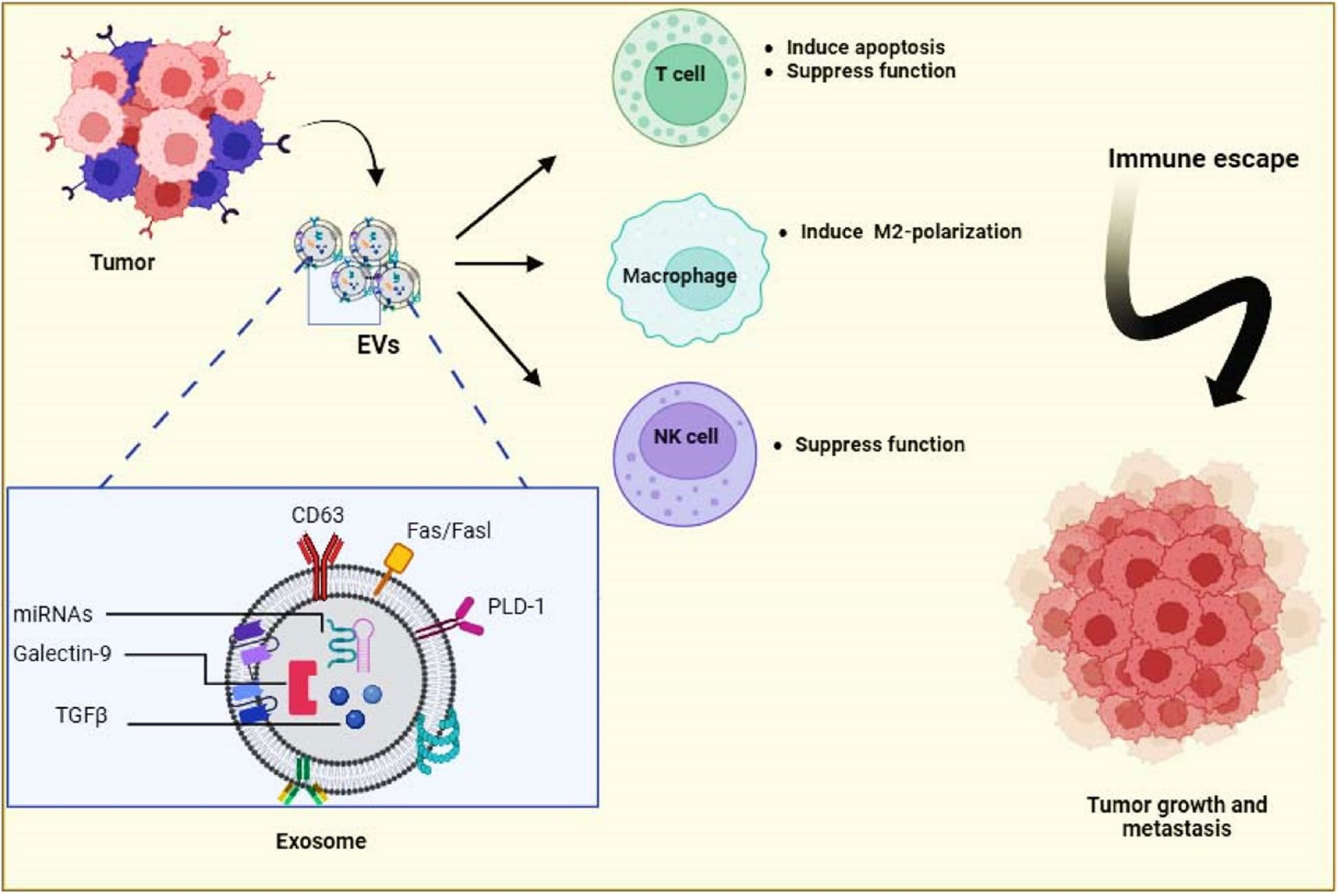
Role of tumor derived EVs in driving immune escape process. Different molecules carried by EVs from tumor cells participate in inducing immune escape

The roles of different EVs from various cancer cells in inducing immune responses have been reported. Tumour cells escape immune identification by increasing the expression levels of PD-L1 that binds to PD-1 receptors on T cells to provoke the immune checkpoint response [51]. This action induces tumor growth. The PD-1/PD-L1 interaction are far more complex. PD-1 have two ligands PD-L1 and PD-L2. PD-L1 is mainly expressed on different cells such as tumor-associated dendritic cells (DCs) [52], macrophages [52], neutrophils [53], monocyte-derived myeloid DCs [54], mast cells [55], fibroblasts [56], and other non-cancerous cells [57]. PD-L2 is expressed in DCs [58] and macrophages [59]. Both PD-L1 and PD-L2 are present in several tumor cells. Recent studies have indicated that EVs-PD-L1 can be more effective than tumor cell-associated PD-L1 in expediting escape from antitumor immunity since EVs can be prevalent in body fluids and may bind to their recipient cells more simply than tumor cells [60]. In glioblastoma cancer, interferon-γ (IFN-γ) stimulated PD-L1 expression on EVs, which inhibited T cell activation. In addition, circulating EVs of glioblastoma patients contain PD-L1 DNA that is correlated with tumor size [61]. EVs from metastatic melanomas have been shown to express PD-L1 on their surface. Several cell culture and animal models showed that exposure to IFN-γ up-regulated the amount of PD-L1 EVs, which inhibited the function of CD8 + T cells and promoted tumor growth. In metastatic melanoma patients, the amount of circulating EVs-PD-L1 is positively associated with that of IFN-γ, and differs following anti-PD-1 therapy [62], Inhibiting the cystine/glutamate transporter cystine-glutamate exchange resulted in higher PD-L1 levels in melanoma and increased EVs-PD-L1 secretion, which in turn induced M2 macrophage polarization and prevented the efficiency of anti-PD-1/PD-L1 therapy in melanoma [63]. The presence of PD-L1 in EVs of human and mouse breast cancer has been described in vitro and in vivo [64]. These EVs repressed the T-cell activation proteins for example CD3/CD28-driven ERK phosphorylation and NF-κB signaling, along with IL-2 secretion. The authors concluded that these EVs could bind to PD-1 and destroy T-cell function, thus inhibiting tumor growth in animal models [64]. The result reported by Chatterjee and co-workers found that TGF-β up-regulated PD-L1 on EVs from breast cancer cells that participated in CD8 T-cell dysfunction by weakening phosphorylation of T-cell receptor (TCR) signaling [65]. Furthermore, in the xenograft mouse model of oral squamous cell carcinoma, mitochondrial Lon-induced EVs containing PD-L1 (EVs-PD-L1) could induce the production of IFN and IL-6 from M2 macrophages, which promoted T-cell dysfunction and tumor progression [66]. Chemotherapies have been shown to induce the production of EVs-PD-L1, which contributes to immunosuppression responses in gastric cancer via the miR-940/Cbl-b/STAT5A axis [67]. In addition, radiotherapy can increase EVs-PDL-1 which promotes immune escape and increases tumor growth [68]. In prostate cancer, Poggio et al. declared that the genetic block of EVs-PD-L1 prolonged survival by endorsing anti-tumor immunity. These EVs suppressed T cell activity in the draining lymph node. They reported that the systemically administration of EVs-PD-L1 rescued the progress of tumors unable to produce their own [69]. Stem cell-derived EVs may participate in immune scape. For example, EVs from mesenchymal stem cells (MSCs) of cancerous mice carry PD-L1 that prevented CD8 + T cells proliferation and activation in experimental models, a role tumor immunosuppression [70] (Fig. 2).

The distinct roles of other tumor derived EVs in immune escape have been prepared in Table 3.

**Table 3:** Role of EVs- PD-L1 in immune escape

Cancer type	Function	Mechanism	Ref
Glioblastoma	Promoted monocytes toward the immune suppressive M2 phenotype and caused immune suppressive function	Up-regulated PD-L1 expression and activated STAT3 pathway	[71]
Breast	Induced an immunosuppressive microenvironment that encourages tumor development	PD-L1 inhibited CD8 + T cells function and polarized macrophages into M2-type	[72]
Gastric	Caused T-cell dysfunction	MHC-I stimulated impaired T cells function	[73]
	Prompt expression of PD-L1 on neutrophils to overturn T-Cell activity	HMGB1 promoted the expression of PD-L1 in neutrophils Triggered STAT3 signaling	[74]
Hepatocellular	Suppressed CD8 + T cells and promoted PD-L1 stabilization	Up-regulated PD-L1 on macrophages by GOLM1	[75]
Head and neck	Impaired the activity of effector T cells	Suppressed CD69 expression	[76]
Non-small cell lung	Induced CD8 + T cells death and tumor development	Suppressed production of IL-2 and IFN-γ by CD8 + T cells/ Reduced number of CD8 + T cells	[77]
	Promoted tumor metastasis	Activated NF-κB signaling and glycolysis dominated metabolic reprogramming pathway that induced the PD-L1 expression level in macrophages	[78]
Lung	Improved tumor growth in vivo	Inhibited cytokine production/ Promoted apoptosis in CD8 + T cells	[60]
Chronic lymphocytic leukemia	Promoted cancer cells escape from antitumor immunity	Induced the PD-L1 levels in macrophages/Increased miR-23a-3p in EVs/ Activated PTEN-AKT axis	[79]
	Inhibited tumor immunity	hY4 in EVs from CLL patients interact with TLR7 on monocytes, thus promoting the expression of inflammatory factors and PD-L1 in monocytes	[80]

### Other molecules

EVs from metastatic oral cancer loaded with HSP90 could induce tumor-associated macrophage (TAM) polarization to an M2 phenotype that promotes tumor development [81]. Head and neck squamous cell carcinoma-derived EVs carry CD73, which supports cancer progression and causes immune evasion [82]. These EVs promoted the activity of NF-κB pathway in TAMs, thus preventing immune responses by promoting cytokines production like TNF-α, IL-10, IL-6, and TGF-β1 [82]. EVs derived from melanoma cells can reach draining lymph nodes and macrophages. These EVs contain tumor antigens that lead to apoptosis in antigen-specific CD8 + T cells and tumor immune inhibition [83]. Melanoma cell-derived EVs stimulate the immunosuppressive functions of MDSCs in regulating T cells. For instance, Andreola et al*.* found that FasL-bearing EVs could stimulate MDSC differentiation through prostaglandin E2 and TGF-β signaling, which lessened MDSC-mediated immunosuppression [84]. They showed that these EVs up-regulated the expression of Cox2, arginase-1, and VEGF in the MDSCs. EVs from two mouse tumor cell lines (the melanoma line MO5 and the thymoma line EG7) expressing the OVA antigen. Participated in prompting tumor antigen-specific immunosuppression, probably by inhibiting DC maturation and modulating the APCs function [85]. EVs from the cerebrospinal fluid of glioblastoma patients carry LGALS9, which could inhibit DCs antigen presentation and T-cell immunity [86]. For hepatocellular carcinoma (HCC), Ye et al*.* reported that HMGB1 from tumor cells promotes immune avoidance of HCC by stimulating TIM-1 + regulatory B cell growth [87]. Recently, it was demonstrated that circGSE1 cargo of EVs of HCC cells increased the development of HCC by prompting Tregs development via inducing the miR-324-5p/TGFBR1/Smad3 signaling. Authors concluded that these EVs can serve as a hopeful biomarker for HCC immunotherapy [88]. TGF-β1 cargo of EVs from pancreatic ductal adenocarcinoma contain molecules that hurt NK cell function by lessening expression of CD107a, NKG2D, INF-γ, and TNF-α, also revealed to damage glucose uptake capacity by NK cells [89]. We presented other studies in Table 4.

**Table 4:** Role of several cargoes of EVs in immune escape

Cancer type	EVs cargo	Function	Mechanism	
Epstein-Barr virus-infected nasopharyngeal carcinoma cells	Galectin-9	Inhibited antitumoral T cell activity	ND	[90]
Human, Squamous cell carcinoma	FasL	Prompted CD8( +) T cell apoptosis	Induced Bax and Bim expression	[91]
Breast	ND	Changed macrophage polarization	Activated gp130/STAT3 signaling pathway	[92]
	ND	Promoted tumor progress and axillary LN metastasis	Prompted M2 polarization	[93]
Murine-derived GL26 cells	ND	Decreased population of CD8 + T cells/ Inhibited CD8 + T cell activity	Apoptosis pathway and inhibiting release of IFN-gamma and granzyme B	[94]
Pleural malignant mesothelioma	TGFβ	Prevented lymphocyte response to Interleukin-2/ Repressed NK cell function	IL-2–mediated CD25 and Foxp3 expression	[95]
HeLa and A375 cells	MICA*008	Reduced NK cytotoxicity/ Induced immune escape	Reduced surface NKG2D receptors	[96]
Ovarian	ND	Induced T cells transform into Treg	Up-regulated the expression of phospho-SMAD2/3 and phospho-STAT3 in Treg	[97]
Hepatocellular carcinoma	miR146a	Induced M2-polarization/ suppressed T cells function	Activated SALL4/miR-146a-5p regulatory axis	[98]
	14–3-3ζ	Impaired anti-tumor function of tumor-infiltrating T cells	Exhausted phenotypes as measured by inhibitory receptors such as PD-1, TIM-3, LAG3, and CTLA-4	[99]
Prostate	FasL	Promoted CD8 + T cell death	Activated FasL-Fas signaling	[100]
	ND	Overturned activity of CD8 + T and NK Cells	Suppressed NKG2D expression	[101]
Gastric	miR-107	Promoted growth of MDSCs	Induced DICER1 and PTEN genes	[102]
Colorectal	FasL/TRAIL	Caused apoptosis in T cells	Delivering FasL and TRAIL, thereby induced apoptosis signaling	[103]

*ND* Not determined

#### EVs-based therapies for overcome immune escape: further directions

**Fig. 3:**
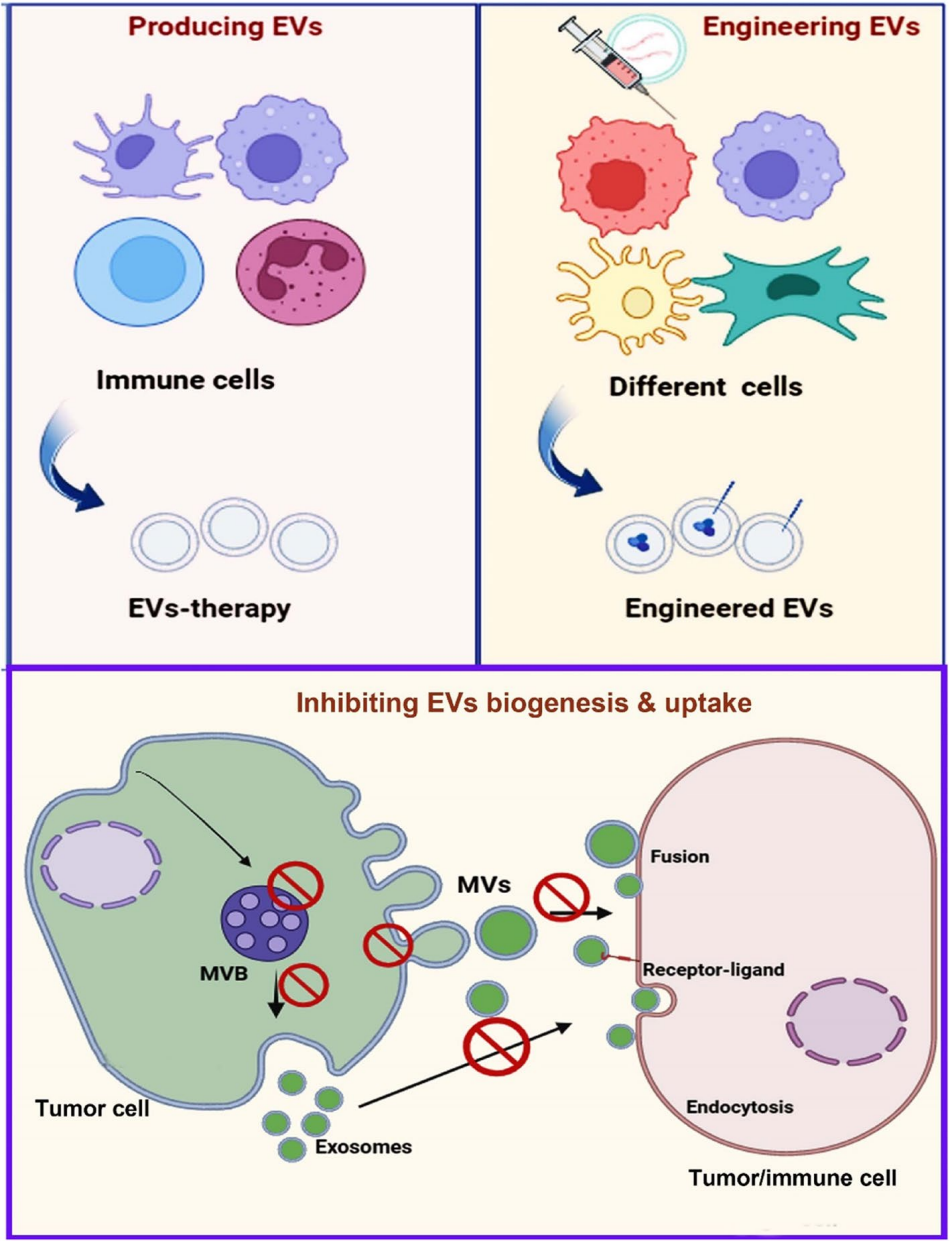
Extracellular vesicles (EVs)-based therapies for overcoming immune escape. To overcome immune escape several EVs-based therapies such as; EVs-therapy from immune cells, inhibiting EVs biogenesis and uptake, and engineering/modifying EVs from different cells (cancer cells, immune cells, and stem cells) have been reported. MVB: multivesicular body; MVs: microvesicles

As mentioned above, EVs released from tumor cells participate in immune escape and immunosuppression, therefore, inhibiting EVs biogenesis, secretion, and internalization may be a possible mechanism for preventing immune evade (for further study see literature [104]) (Fig. 3). Different agents or pharmacological inhibitors may block EVs kinetics [105, 106]. For example, in our recent study, we found that Gallic acid inhibited exosomes biogenesis from two breast cancer cells. We concluded that Gallic acid may serve as an antitumor agent [107]. Reversely, in another study, we found that metformin, an ant-diabetic drug, increased exosomes secretion from glioblastoma cells, suggesting a resistance against therapy [108]. In a study, it was demonstrated that iron death inducer and GW4869 decreased the production of EVs from tumor cells and declined the immunosuppressive impact of EVs-PD-L1 that encouraged anti-cancer immune response of melanoma cells and induced CD + 8 T cells and immune memory [109]. Thus, the evidence from these studies suggests that inhibiting EVs may be a useful approach to overcome immune evade, however, some limitations may remain to be solved. For instance, many of these studies were conducted in vitro comprising cell lines and a low number of animal studies. Therefore, the side effects and systematic toxicity may be associated with these agents. In addition, these agents must only block EVs from cancer cells not from stem or healthy cells. As well, the pharmaceutics of these agents should be determined because EVs biogenesis is cross-talked with other signaling pathways. An inhibition in EVs biogenesis may be compensated with other pathways, causing cancer resistance and bystander effects.

In the exploration for innovative therapeutics, EVs therapies may stand star for overcoming immune escape. The most famous method is using DCs-derived EVs like exosomes for immunotherapy. This method was intensively reviewed in the literature [110, 111], where authors indicated that antigen-loaded exosomes can induce potent antitumor immunity. DCs-derived EVs can both directly and indirectly activate CD + 8 T cells, CD + 4 T cells, NKs, and even B cells for anticancer immunity. Furthermore, DCs-EVs based cancer immunotherapy has been studied in clinical trials [112, 113]. The idea of engaging DC-EVs as an antitumor vaccine approach is using nature’s antigen delivery system for vaccination. Nevertheless, the low clinical efficiency of these vaccines in the stimulation of adaptive immune responses remains a challenge and needs further studies because it seems that the stage of disease and chemotherapy regime are involved in immunotherapy efficacy (Fig. 3).

### Engineered EVs to overcome immune escape

The harnessing of EVs in cancer therapy as a drug delivery system is now being recognized. In this context, EVs are either modified or loaded with optional cargo to overcome tumor expansion and even immune escape. Besides reinforcement, the efficacy of anticancer therapies, engineered EVs, as a novel drug delivery tool, might improve the unwanted effects and side effects of therapies including, radiotherapy and chemotherapy. EVs may genetically be modified or exogenously loaded with therapeutic drugs. However, a survey of literature shows a heterogeneity in both EVs source and engineering methods. However, each engineering technique has its benefits and difficulties and the ‘one-size-fits-all’ engineering method has not been approved yet. For example, recently researchers genetically modified macrophages to overexpress hsa_circ_0004658, which was also carried by their exosomes. When these exosomes co-cultured with HCC cells profoundly inhibited cell growth via miR-499b-5p/JAM3 signaling [33]. Recently, Chen et al*.* engineered an MDA-MB-231 cell line to express a high-affinity mutant human PD-1 protein (havPD-1) and suppress endogenous β-2 microglobulin and PD-L1. These EVs decreased the growth of PD-L1 overexpressed tumor cells and prompted cell death, suggesting a potential for immunotherapy [114]. In pancreatic ductal adenocarcinoma, MSCs-derived EVs were used to carry siRNA and drugs to cancer cells. MSCs-derived EVs containing oxaliplatin (OXA) and galectin-9 siRNA could prompt cell death, and inverse the suppressive tumor immune microenvironment, for instance, preventing polarization of M2 macrophage and the enrolment of T cells, therefore enhancing immunotherapy effectiveness in vitro and in vivo [115]. In an HCC study, EVs were isolated from mouse H22 cells co-cultured with PIONs@E6 and then incubated with macrophages. Findings showed that these EVs promoted immunity against HCC via inducing M1 macrophage polarization and ROS production. Furthermore, PIONs-contained EVs could suppress tumor development in HCC animal model [116]. In pancreatic cancer, Panc-1 cells were loaded with miR-125b2 and miRNA-155 and then EVs were isolated. EVs contain both miRNAs, which could alter the macrophage polarization from M2 to M1 phenotype, favorable for cancer therapy [117]. Table 5 presents the immunological-engineered EVs for cancers. These findings suggest that harnessing engineered EVs showed a hopeful outcome in inducing immune responses and overcoming the immune escape of tumor cells. For clinical translation of these results, further studies are essential.

**Table 5:** Engineered EVs for immunological responses in cancer

EVs source	Target cancer	Cargo	Engineering/loading method	Function	Ref
ReN (Human neural progenitor cell line)	Glioblastoma	RGDyK peptide (RGD/ siRNA against PD-L1	Chemically/ Genetically	Induced CD8 + T cells activity, Suppressed tumor growth and increased animal survival	[118]
M1 macrophage	LLC cells (Mouse lung cancer)	Catalases/ the anti-PD-L1 nanobody/	Chemically/ Incubation	Polarizd M2 macrophages into M1 type, Deceased the immunosuppression of T cells in vitro and in vivo	[119]
	Macrophage/ mice breast cancer	NF-κB siRNA /miR-511–3p/ IL4RPep-1	Genetically/Chemically	Repressed tumor growing, and reduced production of M2 cytokines and immune suppressive cells, Increased M1 cytokines and immune-stimulatory cells	[120]
293T cells (Human embryonic kidney 293 cells)	B-LCLs (B-Lymphoblastoid cell line) and CD8 + T/mice	HPV-E6	Genetically	Induced the activity of CD8 + T Cell, Activated an antigen cross-presentation by DCs	[121]
	In vitro cells/ Mice breast cancer	HER2/neu/Nefmut	Genetically	Promoted CD8 + T activity/ Induced HER2-based CTL responses	[122]
Expi293F (Cells are derived from the 293 cell line)	Breast cancer cells/mice	CD3/EGFR/PD-1/ OX40	Genetically	Caused strong anti-tumor immunity/ Inhibited tumors in mice model	[123]
CAR-T cells	Breast cancer cells/Mice	CAR	Genetically	Increased immune and antitumor responses	[124]
Myeloma cell	Dendritic cells/T cells/Mice	HSP70	Genetically	Promoted maturation of DCs / Induced CD8( +) CTL − and NK-based antitumor immunity	[125]
Pancreatic cancer	Pancreatic cancer	Ce6	Genetically	Augmented the production of cytokines from immune cells and increased immunotherapy	[126]
J558 tumor cells (myeloma cell line)	Mice	TNF-a	Genetically	Induced tumor antigen P1A-specific CD8 + T cells responses	[127]
Muscle cells	Muscle tissues / T cells/ mice lung cancer	Nefmut/E7	Genetically	Induced CD8 + T-cell immune response	[128]
CT26 (Murine colorectal carcinoma cell line), B16-F10 (Murine melanoma cell line), LLC (Mouse lung cancer), and 4T1 (breast cancer cells)	colon, melanoma, lung, breast	α -FAP	Genetically	Induced strong T cells immune response/Promoted the maturation of DCs	[129]
Breast cancer lines	Breast	Human neutrophil elastase (ELANE) and Hiltonol (TLR3 agonist)	Electroporation	Induced ICD in breast cancer cells	[130]
Dendritic cells	Melanoma	Ovalbumin, anti-CD3 and anti-EGFR	Incubation	Increased PD-L1 expression/ Stimulated the T cells growth and activity in vitro and in vivo	[131]
	Hepatocellular carcinoma mice	P47-P/AFP212-A2/N1ND-N	Genetically	Inhibited tumor growth and tumor immunity	[132]
Expi293 (Human cells are derived from the 293 cell line)	Breast	Anti-human HER2 antibodies/ Anti-human CD3 and	Genetically	Promoted anti-tumor activity both in vitro and in vivo	[133]
HEK293 cells (Human Embryonic Kidney cells)	Colorectal cancer and hepatocellular carcinoma	Antisense oligonucleotide (ASO) targeting STAT	Electroporation/ incubation	Induced CD8 + T cell-mediated adaptive immunity	[134]
3LL cells (Murine lung cancer cell line)	Dendritic cells/ Mice lung cancer	TAA, CD40L	Genetically	Promoted CD4 + T cell proliferation /Induced DCs mediated antitumor activity in 3LL tumor	[135]

## Conclusion

Immune escape is a hallmark for tumor development and growth, and may also elucidate the failure of immunotherapy. Tumor cells recruit different mechanism to escape from immune cells, for example, they express PD-L1, which bind to PD-1 on immune cells, thus preventing the T cells function. PD-L1 and other molecules can be transferred by EVs of cancer cells through the biological fluids and cause immunosuppression. Several studies including cell culture and tumor models have shown that EVs from tumor cells containing cargoes like PD-L1 or other molecules play an important function in the immune escape of numerous cancers. These EVs can directly or indirectly suppress several immune cells such as macrophages and T cells. Due to a heterogeneity in EVs types and cargoes, it seems that immune escape elicited by EVs is not simple and different pathways may be involved. EVs-based therapies for overcoming immune escape have been suggested, for example, inhibiting EVs biogenesis and actions. In addition, EVs from immune cells such as DCs or lymphocytes may potent immune responses against tumor cells. Natural EVs may not do effectively on immune responses and even suppress immune cells. EVs could serve as a drug delivery platform for cancer therapy. EVs can be modified or loaded with therapeutic molecules on their cargo or/and on the surface to interact with tumor and immune cells, causing profound antitumor immunity. Several molecules are conjugated into different EVs, which induce T cells and macrophage responses and inhibit tumor growth in preclinical experiments. All EVs-based therapies have several advantages and disadvantages regarding either technical or outcomes. EVs-based clinical application is hindered by the heterogeneity of EVs and the lack of optimized engineering methods.

## Data Availability

None.

## Abbreviations

EVs: Extracellular vesicles
HSPs: Heat shock proteins
PD-L1: Programmed death-ligand 1
PD-1: Programmed death-1
ISEV: International Society for Extracellular Vesicles
MISEV: Minimal Information for Studies of Extracellular vesicles
T-EVs: Tumor-derived extracellular vesicles
MVB: Multivesicular bodies
ILVs: Intraluminal vesicles
DCs: Dendritic cells
IFN-γ: Interferon-γ
TCR: T-cell receptor
MSCs: Mesenchymal stem cells
MHC- 1: Major histocompatibility complex 1
HMGB1: High mobility group box 1
HCC: Hepatocellular carcinoma
TAM: Tumor-associated macrophage
MDSCs: Myeloid-derived suppressor cells
TGF-β: Transforming Growth Factor β
OVA: Ovalbumin
VEGF: Vascular Endothelial Growth Factor
APCs: Antigen-presenting cells
LGALS9: Galectin9
ICD: Immunogenic cell death

## References

[1] Beatty GL, Gladney WL (2015). Immune escape mechanisms as a guide for cancer immunotherapy. Clin Cancer Res.

[2] Liu Y, Cao X (2016). Immunosuppressive cells in tumor immune escape and metastasis. J Mol Med.

[3] Ri MH, Ma J, Jin X (2021). Development of natural products for anti-PD-1/PD-L1 immunotherapy against cancer. J Ethnopharmacol.

[4] Ishikawa M, Nakayama K, Nakamura K, Yamashita H, Ishibashi T, Minamoto T, Iida K, Razia S, Ishikawa N, Nakayama S (2020). High PD-1 expression level is associated with an unfavorable prognosis in patients with cervical adenocarcinoma. Arch Gynecol Obstet.

[5] Babaei M, Pirnejad H, Rezaie J, Roshandel G, Hoseini R (2023). Association between socioeconomic factors and the risk of gastric cancer incidence: results from an ecological study. Iran J Public Health.

[6] Babaei M, Hasanzadeh S, Pirnejad H, Mohebbi I, Hoseini R, Niazkhani Z (2022). Socioeconomic status and severity of traffic accident injuries: a cross-sectional study. Iran Occupational Health.

[7] Rezaie J, Etemadi T, Feghhi M. The distinct roles of exosomes in innate immune responses and therapeutic applications in cancer. Eur J Pharmacol. 2022:933:175292. 10.1016/j.ejphar.2022.175292 36150532

[8] Van Niel G, d'Angelo G, Raposo G (2018). Shedding light on the cell biology of extracellular vesicles. Nat Rev Mol Cell Biol.

[9] Feghhi M, Rezaie J, Akbari A, Jabbari N, Jafari H, Seidi F, Szafert S (2021). Effect of multi-functional polyhydroxylated polyhedral oligomeric silsesquioxane (POSS) nanoparticles on the angiogenesis and exosome biogenesis in human umbilical vein endothelial cells (HUVECs). Mater Des.

[10] van de Wakker SI, Meijers FM, Sluijter JPG, Vader P (2023). Extracellular vesicle heterogeneity and its impact for regenerative medicine applications. Pharmacol Rev.

[11] Théry C, Witwer KW, Aikawa E, Alcaraz MJ, Anderson JD, Andriantsitohaina R, Antoniou A, Arab T, Archer F, Atkin-Smith GK (2018). Minimal information for studies of extracellular vesicles 2018 (MISEV2018): a position statement of the International Society for Extracellular Vesicles and update of the MISEV2014 guidelines. J Extracell Vesicles.

[12] Lötvall J, Hill AF, Hochberg F, Buzás EI, Di Vizio D, Gardiner C, Gho YS, Kurochkin IV, Mathivanan S, Quesenberry P: Minimal experimental requirements for definition of extracellular vesicles and their functions: a position statement from the International Society for Extracellular Vesicles. vol. 3. Taylor & Francis; 2014:26913. 10.3402/jev.v3.26913 PMC4275645 25536934

[13] Taghikhani A, Farzaneh F, Sharifzad F, Mardpour S, Ebrahimi M, Hassan ZM (2020). Engineered tumor-derived extracellular vesicles: potentials in cancer immunotherapy. Front Immunol.

[14] Ma J, Zhang H, Tang K, Huang B (2020). Tumor-derived microparticles in tumor immunology and immunotherapy. Eur J Immunol.

[15] Wu F, Gu Y, Kang B, Heskia F, Pachot A, Bonneville M, Wei P, Liang J (2021). PD-L1 detection on circulating tumor-derived extracellular vesicles (T-EVs) from patients with lung cancer. Transl Lung Cancer Res.

[16] Su D, Tsai H-I, Xu Z, Yan F, Wu Y, Xiao Y, Liu X, Wu Y, Parvanian S, Zhu W (2020). Exosomal PD-L1 functions as an immunosuppressant to promote wound healing. J Extracell Vesicles.

[17] Martin P, Leibovich SJ (2005). Inflammatory cells during wound repair: the good, the bad and the ugly. Trends Cell Biol.

[18] Czystowska-Kuzmicz M, Sosnowska A, Nowis D, Ramji K, Szajnik M, Chlebowska-Tuz J, Wolinska E, Gaj P, Grazul M, Pilch Z (2019). Small extracellular vesicles containing arginase-1 suppress T-cell responses and promote tumor growth in ovarian carcinoma. Nat Commun.

[19] Mincheva-Nilsson L, Baranov V: Cancer exosomes and NKG2D receptor–ligand interactions: Impairing NKG2D-mediated cytotoxicity and anti-tumour immune surveillance. In Seminars in Cancer Biology. Elsevier; 2014: 24–30. 10.1016/j.semcancer.2014.02.010 24602822

[20] Zhang H, Lu J, Liu J, Zhang G, Lu A (2020). Advances in the discovery of exosome inhibitors in cancer. J Enzyme Inhib Med Chem.

[21] Yao Y, Fu C, Zhou L, Mi Q-S, Jiang A (2021). DC-derived exosomes for cancer immunotherapy. Cancers.

[22] Liu C, Wang Y, Li L, He D, Chi J, Li Q, Wu Y, Zhao Y, Zhang S, Wang L (2022). Engineered extracellular vesicles and their mimetics for cancer immunotherapy. J Control Release.

[23] Rezaie J, Nejati V, Mahmoodi M, Ahmadi M. Mesenchymal stem cells derived extracellular vesicles: a promising nanomedicine for drug delivery system. Biochem Pharmacol. 2022:203:115167. 10.1016/j.bcp.2022.115167 35820499

[24] Ahmadi M, Hassanpour M, Rezaie J. Engineered extracellular vesicles: a novel platform for cancer combination therapy and cancer immunotherapy. Life Sci. 2022:308:120935. 10.1016/j.lfs.2022.120935 36075472

[25] Hassanzadeh A, Ashrafihelan J, Salehi R, Rahbarghazi R, Firouzamandi M, Ahmadi M, Khaksar M, Alipour M, Aghazadeh M (2021). Development and biocompatibility of the injectable collagen/nano-hydroxyapatite scaffolds as in situ forming hydrogel for the hard tissue engineering application. Artif Cells Nanomed Biotechnol.

[26] Raposo G, Stahl PD (2019). Extracellular vesicles: a new communication paradigm?. Nat Rev Mol Cell Biol.

[27] Bewicke-Copley F, Mulcahy LA, Jacobs LA, Samuel P, Akbar N, Pink RC, Carter DRF (2017). Extracellular vesicles released following heat stress induce bystander effect in unstressed populations. J Extracell Vesicles.

[28] Takahashi A, Okada R, Nagao K, Kawamata Y, Hanyu A, Yoshimoto S, Takasugi M, Watanabe S, Kanemaki MT, Obuse C (2017). Exosomes maintain cellular homeostasis by excreting harmful DNA from cells. Nat Commun.

[29] Hassanpour M, Rezaie J, Darabi M, Hiradfar A, Rahbarghazi R, Nouri M (2020). Autophagy modulation altered differentiation capacity of CD146+ cells toward endothelial cells, pericytes, and cardiomyocytes. Stem Cell Res Ther.

[30] Baixauli F, López-Otín C, Mittelbrunn M (2014). Exosomes and autophagy: coordinated mechanisms for the maintenance of cellular fitness. Front Immunol.

[31] Mulcahy LA, Pink RC, Carter DRF (2014). Routes and mechanisms of extracellular vesicle uptake. J Extracell Vesicles.

[32] French KC, Antonyak MA, Cerione RA. Extracellular vesicle docking at the cellular port: Extracellular vesicle binding and uptake. In Seminars in cell & developmental biology. Elsevier; 2017:67:48–55. 10.1016/j.semcdb.2017.01.002 PMC5484727 28104520

[33] Zhang L, Zhang J, Li P, Li T, Zhou Z, Wu H (2022). Exosomal hsa_circ_0004658 derived from RBPJ overexpressed-macrophages inhibits hepatocellular carcinoma progression via miR-499b-5p/JAM3. Cell Death Dis.

[34] Buzas EI (2023). The roles of extracellular vesicles in the immune system. Nat Rev Immunol.

[35] Hessvik NP, Llorente A (2018). Current knowledge on exosome biogenesis and release. Cell Mol Life Sci.

[36] Fader C, Colombo M (2009). Autophagy and multivesicular bodies: two closely related partners. Cell Death Differ.

[37] Jeppesen DK, Fenix AM, Franklin JL, Higginbotham JN, Zhang Q, Zimmerman LJ, Liebler DC, Ping J, Liu Q, Evans R (2019). Reassessment of exosome composition. Cell.

[38] Buschow SI, Nolte-‘t Hoen EN, Van Niel G, Pols MS, Ten Broeke T, Lauwen M, Ossendorp F, Melief CJ, Raposo G, Wubbolts R (2009). MHC II in dendritic cells is targeted to lysosomes or T cell-induced exosomes via distinct multivesicular body pathways. Traffic.

[39] Klumperman J, Raposo G (2014). The complex ultrastructure of the endolysosomal system. Cold Spring Harb Perspect Biol.

[40] Villarroya-Beltri C, Baixauli F, Mittelbrunn M, Fernández-Delgado I, Torralba D, Moreno-Gonzalo O, Baldanta S, Enrich C, Guerra S, Sánchez-Madrid F (2016). ISGylation controls exosome secretion by promoting lysosomal degradation of MVB proteins. Nat Commun.

[41] Ortega FG, Roefs MT, de Miguel Perez D, Kooijmans SA, de Jong OG, Sluijter JP, Schiffelers RM, Vader P (2019). Interfering with endolysosomal trafficking enhances release of bioactive exosomes. Nanomed Nanotechnol Biol Med.

[42] Zabeo D, Cvjetkovic A, Lässer C, Schorb M, Lötvall J, Höög JL (2017). Exosomes purified from a single cell type have diverse morphology. J Extracell Vesicles.

[43] Mathieu M, Névo N, Jouve M, Valenzuela JI, Maurin M, Verweij FJ, Palmulli R, Lankar D, Dingli F, Loew D (2021). Specificities of exosome versus small ectosome secretion revealed by live intracellular tracking of CD63 and CD9. Nat Commun.

[44] Andreu Z, Yáñez-Mó M (2014). Tetraspanins in extracellular vesicle formation and function. Front Immunol.

[45] Tricarico C, Clancy J, D'Souza-Schorey C (2017). Biology and biogenesis of shed microvesicles. Small GTPases.

[46] Willms E, Cabañas C, Mäger I, Wood MJ, Vader P (2018). Extracellular vesicle heterogeneity: subpopulations, isolation techniques, and diverse functions in cancer progression. Front Immunol.

[47] Whiteside T (2017). Exosomes carrying immunoinhibitory proteins and their role in cancer. Clin Exp Immunol.

[48] Inamdar S, Nitiyanandan R, Rege K (2017). Emerging applications of exosomes in cancer therapeutics and diagnostics. Bioeng Transl Med.

[49] Yáñez-Mó M, Siljander PR-M, Andreu Z, Bedina Zavec A, Borràs FE, Buzas EI, Buzas K, Casal E, Cappello F, Carvalho J (2015). Biological properties of extracellular vesicles and their physiological functions. J Extracell Vesicles.

[50] Hassani A, Avci ÇB, Kerdar SN, Amini H, Amini M, Ahmadi M, Sakai S, Bagca BG, Ozates NP, Rahbarghazi R (2022). Interaction of alginate with nano-hydroxyapatite-collagen using strontium provides suitable osteogenic platform. J Nanobiotechnol.

[51] Litak J, Mazurek M, Grochowski C, Kamieniak P, Roliński J (2019). PD-L1/PD-1 axis in glioblastoma multiforme. Int J Mol Sci.

[52] Peng Q, Qiu X, Zhang Z, Zhang S, Zhang Y, Liang Y, Guo J, Peng H, Chen M, Fu Y-X (2020). PD-L1 on dendritic cells attenuates T cell activation and regulates response to immune checkpoint blockade. Nat Commun.

[53] Cheng Y, Li H, Deng Y, Tai Y, Zeng K, Zhang Y, Liu W, Zhang Q, Yang Y. Cancer-associated fibroblasts induce PDL1+ neutrophils through the IL6-STAT3 pathway that foster immune suppression in hepatocellular carcinoma. Cell Death Dis. 2018;9:422. 10.1038/s41419-018-0458-4 PMC5859264 29556041

[54] Curiel TJ, Wei S, Dong H, Alvarez X, Cheng P, Mottram P, Krzysiek R, Knutson KL, Daniel B, Zimmermann MC. Blockade of B7–H1 improves myeloid dendritic cell-mediated antitumor immunity. Nat Med. 2003;9:562–7. 10.1038/nm863 12704383

[55] Hirano T, Honda T, Kanameishi S, Honda Y, Egawa G, Kitoh A, Nakajima S, Otsuka A, Nomura T, Dainichi T. PD-L1 on mast cells suppresses effector CD8(+) T-cell activation in the skin in murine contact hypersensitivity. J Allergy Clin Immunol. 2021;148:563–73. 10.1016/j.jaci.2020.12.654 33581199

[56] Teramoto K, Igarashi T, Kataoka Y, Ishida M, Hanaoka J, Sumimoto H, Daigo Y. Clinical significance of PD-L1-positive cancer-associated fibroblasts in pN0M0 non-small cell lung cancer. Lung Cancer. 2019;137:56–63. 10.1016/j.lungcan.2019.09.013 31546072

[57] Sun C, Mezzadra R, Schumacher TN. Regulation and function of the PD-L1 checkpoint. Immunity. 2018;48:434–52. 10.1016/j.immuni.2018.03.014 PMC7116507 29562194

[58] Garo LP, Ajay AK, Fujiwara M, Beynon V, Kuhn C, Gabriely G, Sadhukan S, Raheja R, Rubino S, Weiner HL, Murugaiyan G. Smad7 controls immunoregulatory PDL2/1-PD1 signaling in intestinal inflammation and autoimmunity. Cell Rep. 2019;28:3353–66. 10.1016/j.celrep.2019.07.065 PMC6925592 31553906

[59] Loke P, Allison JP: PD-L1 and PD-L2 are differentially regulated by Th1 and Th2 cells. Proc Natl Acad Sci USA. 2003;100:5336–41. 10.1073/pnas.0931259100 PMC154346 12697896

[60] Kim DH, Kim H, Choi YJ, Kim SY, Lee J-E, Sung KJ, Sung YH, Pack C-G, Jung M-K, Han B (2019). Exosomal PD-L1 promotes tumor growth through immune escape in non-small cell lung cancer. Exper Mol Med.

[61] Ricklefs FL, Alayo Q, Krenzlin H, Mahmoud AB, Speranza MC, Nakashima H, Hayes JL, Lee K, Balaj L, Passaro C. Immune evasion mediated by PD-L1 on glioblastoma-derived extracellular vesicles. Sci Adv. 2018;4:eaar2766. 10.1126/sciadv.aar2766 PMC5842038 29532035

[62] Chen G, Huang AC, Zhang W, Zhang G, Wu M, Xu W, Yu Z, Yang J, Wang B, Sun H (2018). Exosomal PD-L1 contributes to immunosuppression and is associated with anti-PD-1 response. Nature.

[63] Liu N, Zhang J, Yin M, Liu H, Zhang X, Li J, Yan B, Guo Y, Zhou J, Tao J (2021). Inhibition of xCT suppresses the efficacy of anti-PD-1/L1 melanoma treatment through exosomal PD-L1-induced macrophage M2 polarization. Mol Ther.

[64] Yang Y, Li CW, Chan LC, Wei Y, Hsu JM, Xia W, Cha JH, Hou J, Hsu JL, Sun L, Hung MC. Exosomal PD-L1 harbors active defense function to suppress T cell killing of breast cancer cells and promote tumor growth. Cell Res. 2018;28:862–4. 10.1038/s41422-018-0060-4 PMC6082826 29959401

[65] Chatterjee S, Chatterjee A, Jana S, Dey S, Roy H, Das MK, Alam J, Adhikary A, Chowdhury A, Biswas A. Transforming growth factor beta orchestrates PD-L1 enrichment in tumor-derived exosomes and mediates CD8 T-cell dysfunction regulating early phosphorylation of TCR signalome in breast cancer. Carcinogenesis. 2021;42:38–47. 10.1093/carcin/bgaa092 32832992

[66] Cheng AN, Cheng LC, Kuo CL, Lo YK, Chou HY, Chen CH, Wang YH, Chuang TH, Cheng SJ, Lee AY. Mitochondrial Lon-induced mtDNA leakage contributes to PD-L1-mediated immunoescape via STING-IFN signaling and extracellular vesicles. J Immunother Cancer. 2020;8:e001372. 10.1136/jitc-2020-001372 PMC7713199 33268351

[67] Zhang M, Fan Y, Che X, Hou K, Zhang C, Li C, Wen T, Wang S, Cheng Y, Liu Y, Qu X. 5-FU-induced upregulation of exosomal PD-L1 causes immunosuppression in advanced gastric cancer patients. Front Oncol. 2020;10:492. 10.3389/fonc.2020.00492 PMC7188923 32391259

[68] Timaner M, Kotsofruk R, Raviv Z, Magidey K, Shechter D, Kan T, Nevelsky A, Daniel S, Vries EGE, Zhang T. Microparticles from tumors exposed to radiation promote immune evasion in part by PD-L1. Oncogene. 2020;39:187–203. 10.1038/s41388-019-0971-7 PMC6937213 31467431

[69] Poggio M, Hu T, Pai CC, Chu B, Belair CD, Chang A, Montabana E, Lang UE, Fu Q, Fong L, Blelloch R. Suppression of exosomal PD-L1 Induces systemic anti-tumor immunity and memory. Cell. 2019;177:414–27. 10.1016/j.cell.2019.02.016 PMC6499401 30951669

[70] Sun Y, Guo J, Yu L, Guo T, Wang J, Wang X, Chen Y. PD-L1(+) exosomes from bone marrow-derived cells of tumor-bearing mice inhibit antitumor immunity. Cell Mol Immunol. 2020;18:2402–9. 10.1038/s41423-020-0487-7 PMC8484656 32606317

[71] Gabrusiewicz K, Li X, Wei J, Hashimoto Y, Marisetty AL, Ott M, Wang F, Hawke D, Yu J, Healy LM. Glioblastoma stem cell-derived exosomes induce M2 macrophages and PD-L1 expression on human monocytes. Oncoimmunology. 2018;7:e1412909. 10.1080/2162402X.2017.1412909 PMC5889290 29632728

[72] Li C, Qiu S, Jin K, Zheng X, Zhou X, Jin D, Xu B, Jin X. Tumor-derived microparticles promote the progression of triple-negative breast cancer via PD-L1-associated immune suppression. Cancer Lett. 2021;523:43–56. 10.1016/j.canlet.2021.09.039 34601021

[73] Fan Y, Che X, Qu J, Hou K, Wen T, Li Z, Li C, Wang S, Xu L, Liu Y, Qu X. Exosomal PD-L1 retains immunosuppressive activity and is associated with gastric cancer prognosis. Ann Surg Oncol. 2019;26:3745–55. 10.1245/s10434-019-07431-7 31087180

[74] Shi Y, Zhang J, Mao Z, Jiang H, Liu W, Shi H, Ji R, Xu W, Qian H, Zhang X. Extracellular vesicles from gastric cancer cells induce PD-L1 expression on neutrophils to suppress T-Cell immunity. Front Oncol. 2020;10:629. 10.3389/fonc.2020.00629 PMC7237746 32477934

[75] Chen J, Lin Z, Liu L, Zhang R, Geng Y, Fan M, Zhu W, Lu M, Lu L, Jia H (2021). GOLM1 exacerbates CD8+ T cell suppression in hepatocellular carcinoma by promoting exosomal PD-L1 transport into tumor-associated macrophages. Signal Transduct Target Ther.

[76] Theodoraki MN, Yerneni SS, Hoffmann TK, Gooding WE, Whiteside TL. Clinical significance of PD-L1(+) exosomes in plasma of head and neck cancer patients. Clin Cancer Res. 2018;24:896–905. 10.1158/1078-0432.CCR-17-2664 PMC6126905 29233903

[77] Liu D, Wang S, Bindeman W. Clinical applications of PD-L1 bioassays for cancer immunotherapy. J Hematol Oncol. 2017;10:1–6. 10.1186/s13045-017-0479-y PMC5436438 28514966

[78] Morrissey SM, Zhang F, Ding C, Montoya-Durango DE, Hu X, Yang C, Wang Z, Yuan F, Fox M, Zhang HG. Tumor-derived exosomes drive immunosuppressive macrophages in a pre-metastatic niche through glycolytic dominant metabolic reprogramming. Cell Metab. 2021;33:2040–58. 10.1016/j.cmet.2021.09.002 PMC8506837 34559989

[79] Liu J, Fan L, Yu H, Zhang J, He Y, Feng D, Wang F, Li X, Liu Q, Li Y. Endoplasmic reticulum stress causes liver cancer cells to release exosomal miR-23a-3p and up-regulate programmed death ligand 1 expression in macrophages. Hepatology. 2019;70:241–58. 10.1002/hep.30607 PMC6597282 30854665

[80] Haderk F, Schulz R, Iskar M, Cid LL, Worst T, Willmund KV, Schulz A, Warnken U, Seiler J, Benner A. Tumor-derived exosomes modulate PD-L1 expression in monocytes. Sci Immunol. 2017;2:eaah5509. 10.1126/sciimmunol.aah5509 28754746

[81] Ono K, Sogawa C, Kawai H, Tran MT, Taha EA, Lu Y, Oo MW, Okusha Y, Okamura H, Ibaragi S (2020). Triple knockdown of CDC37, HSP90-alpha and HSP90-beta diminishes extracellular vesicles-driven malignancy events and macrophage M2 polarization in oral cancer. J Extracell Vesicles.

[82] Lu T, Zhang Z, Zhang J, Pan X, Zhu X, Wang X, Li Z, Ruan M, Li H, Chen W, Yan M (2022). CD73 in small extracellular vesicles derived from HNSCC defines tumour-associated immunosuppression mediated by macrophages in the microenvironment. J Extracell Vesicles.

[83] Leary N, Walser S, He Y, Cousin N, Pereira P, Gallo A, Collado-Diaz V, Halin C, Garcia-Silva S, Peinado H, Dieterich LC (2022). Melanoma-derived extracellular vesicles mediate lymphatic remodelling and impair tumour immunity in draining lymph nodes. J Extracell Vesicles.

[84] Andreola G, Rivoltini L, Castelli C, Huber V, Perego P, Deho P, Squarcina P, Accornero P, Lozupone F, Lugini L (2002). Induction of lymphocyte apoptosis by tumor cell secretion of FasL-bearing microvesicles. J Exp Med.

[85] Yang C, Kim S-H, Bianco NR, Robbins PD (2011). Tumor-derived exosomes confer antigen-specific immunosuppression in a murine delayed-type hypersensitivity model. PLoS ONE.

[86] Wang M, Cai Y, Peng Y, Xu B, Hui W, Jiang Y (2020). Exosomal LGALS9 in the cerebrospinal fluid of glioblastoma patients suppressed dendritic cell antigen presentation and cytotoxic T-cell immunity. Cell Death Dis.

[87] Ye L, Zhang Q, Cheng Y, Chen X, Wang G, Shi M, Zhang T, Cao Y, Pan H, Zhang L (2018). Tumor-derived exosomal HMGB1 fosters hepatocellular carcinoma immune evasion by promoting TIM-1+ regulatory B cell expansion. J Immunother Cancer.

[88] Huang M, Huang X, Huang N (2022). Exosomal circGSE1 promotes immune escape of hepatocellular carcinoma by inducing the expansion of regulatory T cells. Cancer Sci.

[89] Zhao J, Schlößer HA, Wang Z, Qin J, Li J, Popp F, Popp MC, Alakus H, Chon S-H, Hansen HP (2019). Tumor-derived extracellular vesicles inhibit natural killer cell function in pancreatic cancer. Cancers.

[90] Klibi J, Niki T, Riedel A, Pioche-Durieu C, Souquere S, Rubinstein E, Le Moulec S, Guigay J, Hirashima M, Guemira F (2009). Blood diffusion and Th1-suppressive effects of galectin-9–containing exosomes released by Epstein-Barr virus–infected nasopharyngeal carcinoma cells. Blood J Am Soc Hematol.

[91] Czystowska M, Han J, Szczepanski MJ, Szajnik M, Quadrini K, Brandwein H, Hadden JW, Signorelli K, Whiteside TL (2009). IRX-2, a novel immunotherapeutic, protects human T cells from tumor-induced cell death. Cell Death Differ.

[92] Ham S, Lima LG, Chai EPZ, Muller A, Lobb RJ, Krumeich S, Wen SW, Wiegmans AP, Möller A (2018). Breast cancer-derived exosomes alter macrophage polarization via gp130/STAT3 signaling. Front Immunol.

[93] Piao YJ, Kim HS, Hwang EH, Woo J, Zhang M, Moon WK (2018). Breast cancer cell-derived exosomes and macrophage polarization are associated with lymph node metastasis. Oncotarget.

[94] Liu Z-M, Wang Y-B, Yuan X-H (2013). Exosomes from murine-derived GL26 cells promote glioblastoma tumor growth by reducing number and function of CD8+ T cells. Asian Pac J Cancer Prev.

[95] Clayton A, Mitchell JP, Court J, Mason MD, Tabi Z (2007). Human tumor-derived exosomes selectively impair lymphocyte responses to Interleukin-2. Can Res.

[96] Ashiru O, Boutet P, Fernández-Messina L, Agüera-González S, Skepper JN, Valés-Gómez M, Reyburn HT (2010). Natural killer cell cytotoxicity is suppressed by exposure to the human NKG2D ligand MICA*008 that is shed by tumor cells in exosomes. Can Res.

[97] Szajnik M, Czystowska M, Szczepanski MJ, Mandapathil M, Whiteside TL (2010). Tumor-derived microvesicles induce, expand and up-regulate biological activities of human regulatory T cells (treg). PLoS ONE.

[98] Yin C, Han Q, Xu D, Zheng B, Zhao X, Zhang J (2019). SALL4-mediated upregulation of exosomal miR-146a-5p drives T-cell exhaustion by M2 tumor-associated macrophages in HCC. Oncoimmunology.

[99] Wang X, Shen H, Zhangyuan G, Huang R, Zhang W, He Q, Jin K, Zhuo H, Zhang Z, Wang J (2018). 14-3-3ζ delivered by hepatocellular carcinoma-derived exosomes impaired anti-tumor function of tumor-infiltrating T lymphocytes. Cell Death Dis.

[100] Abusamra AJ, Zhong Z, Zheng X, Li M, Ichim TE, Chin JL, Min W-P (2005). Tumor exosomes expressing Fas ligand mediate CD8+ T-cell apoptosis. Blood Cells Mol Dis.

[101] Lundholm M, Schröder M, Nagaeva O, Baranov V, Widmark A, Mincheva-Nilsson L, Wikström P (2014). Prostate tumor-derived exosomes down-regulate NKG2D expression on natural killer cells and CD8+ T cells: mechanism of immune evasion. PLoS ONE.

[102] Ren W, Zhang X, Li W, Feng Q, Feng H, Tong Y, Rong H, Wang W, Zhang D, Zhang Z. Exosomal miRNA-107 induces myeloid-derived suppressor cell expansion in gastric cancer. Cancer Manag Res. 2019:11:4023–40. 10.2147/CMAR.S198886 PMC6511657 31190980

[103] Huber V, Fais S, Iero M, Lugini L, Canese P, Squarcina P, Zaccheddu A, Colone M, Arancia G, Gentile M (2005). Human colorectal cancer cells induce T-cell death through release of proapoptotic microvesicles: role in immune escape. Gastroenterology.

[104] Catalano M, O’Driscoll L (2020). Inhibiting extracellular vesicles formation and release: a review of EV inhibitors. J Extracell Vesicles.

[105] Atanassoff AP, Wolfmeier H, Schoenauer R, Hostettler A, Ring A, Draeger A, Babiychuk EB (2014). Microvesicle shedding and lysosomal repair fulfill divergent cellular needs during the repair of streptolysin O-induced plasmalemmal damage. PLoS ONE.

[106] Zhou X, Zhang W, Yao Q, Zhang H, Dong G, Zhang M, Liu Y, Chen J-K, Dong Z (2017). Exosome production and its regulation of EGFR during wound healing in renal tubular cells. Am J Physiol-Renal Physiol.

[107] Jabbari N, Feghhi M, Esnaashari O, Soraya H, Rezaie J (2022). Inhibitory effects of gallic acid on the activity of exosomal secretory pathway in breast cancer cell lines: A possible anticancer impact. BioImpacts BI.

[108] Soraya H, Sani NA, Jabbari N, Rezaie J (2021). Metformin increases exosome biogenesis and secretion in U87 MG human glioblastoma cells: a possible mechanism of therapeutic resistance. Arch Med Res.

[109] Wang G, Xie L, Li B, Sang W, Yan J, Li J, Tian H, Li W, Zhang Z, Tian Y (2021). A nanounit strategy reverses immune suppression of exosomal PD-L1 and is associated with enhanced ferroptosis. Nat Commun.

[110] Ghorbaninezhad F, Alemohammad H, Najafzadeh B, Masoumi J, Shadbad MA, Shahpouri M, Saeedi H, Rahbarfarzam O, Baradaran B (2023). Dendritic cell-derived exosomes: a new horizon in personalized cancer immunotherapy?. Cancer Lett.

[111] Viaud S, Théry C, Ploix S, Tursz T, Lapierre V, Lantz O, Zitvogel L, Chaput N (2010). Dendritic cell-derived exosomes for cancer immunotherapy: what's next?. Can Res.

[112] Morse MA, Garst J, Osada T, Khan S, Hobeika A, Clay TM, Valente N, Shreeniwas R, Sutton MA, Delcayre A (2005). A phase I study of dexosome immunotherapy in patients with advanced non-small cell lung cancer. J Transl Med.

[113] Besse B, Charrier M, Lapierre V, Dansin E, Lantz O, Planchard D, Le Chevalier T, Livartoski A, Barlesi F, Laplanche A (2016). Dendritic cell-derived exosomes as maintenance immunotherapy after first line chemotherapy in NSCLC. Oncoimmunology.

[114] Chen Y, Wang L, Zheng M, Zhu C, Wang G, Xia Y, Blumenthal EJ, Mao W, Wan Y (2022). Engineered extracellular vesicles for concurrent anti-PDL1 immunotherapy and chemotherapy. Bioactive materials.

[115] Zhou W, Zhou Y, Chen X, Ning T, Chen H, Guo Q, Zhang Y, Liu P, Zhang Y, Li C (2021). Pancreatic cancer-targeting exosomes for enhancing immunotherapy and reprogramming tumor microenvironment. Biomaterials.

[116] Chen H, Jiang S, Zhang P, Ren Z, Wen J (2021). Exosomes synergized with PIONs@E6 enhance their immunity against hepatocellular carcinoma via promoting M1 macrophages polarization. Int Immunopharmacol.

[117] Su M-J, Aldawsari H, Amiji M (2016). Pancreatic cancer cell exosome-mediated macrophage reprogramming and the role of MicroRNAs 155 and 125b2 transfection using nanoparticle delivery systems. Sci Rep.

[118] Tian T, Liang R, Erel-Akbaba G, Saad L, Obeid PJ, Gao J, Chiocca EA, Weissleder R, Tannous BA (2022). Immune checkpoint inhibition in GBM primed with radiation by engineered extracellular vesicles. ACS Nano.

[119] Ma X, Yao M, Gao Y, Yue Y, Li Y, Zhang T, Nie G, Zhao X, Liang X (2022). Functional immune cell-derived exosomes engineered for the trilogy of radiotherapy sensitization. Advanced Science.

[120] Gunassekaran GR, Poongkavithai Vadevoo SM, Baek M-C, Lee B (2021). M1 macrophage exosomes engineered to foster M1 polarization and target the IL-4 receptor inhibit tumor growth by reprogramming tumor-associated macrophages into M1-like macrophages. Biomaterials.

[121] Manfredi F, Di Bonito P, Ridolfi B, Anticoli S, Arenaccio C, Chiozzini C, Baz Morelli A, Federico M (2016). The CD8+ T cell-mediated immunity induced by HPV-E6 uploaded in engineered exosomes is improved by ISCOMATRIXTM adjuvant. Vaccines.

[122] Anticoli S, Aricò E, Arenaccio C, Manfredi F, Chiozzini C, Olivetta E, Ferrantelli F, Lattanzi L, D’Urso MT, Proietti E, Federico M (2018). Engineered exosomes emerging from muscle cells break immune tolerance to HER2 in transgenic mice and induce antigen-specific CTLs upon challenge by human dendritic cells. J Mol Med.

[123] Cheng Q, Dai Z, Smbatyan G, Epstein AL, Lenz H-J, Zhang Y (2022). Eliciting anti-cancer immunity by genetically engineered multifunctional exosomes. Mol Ther.

[124] Fu W, Lei C, Liu S, Cui Y, Wang C, Qian K, Li T, Shen Y, Fan X, Lin F (2019). CAR exosomes derived from effector CAR-T cells have potent antitumour effects and low toxicity. Nat Commun.

[125] Xie Y, Bai O, Zhang H, Yuan J, Zong S, Chibbar R, Slattery K, Qureshi M, Wei Y, Deng Y, Xiang J (2010). Membrane-bound HSP70-engineered myeloma cell-derived exosomes stimulate more efficient CD8+ CTL- and NK-mediated antitumour immunity than exosomes released from heat-shocked tumour cells expressing cytoplasmic HSP70. J Cell Mol Med.

[126] Jang Y, Kim H, Yoon S, Lee H, Hwang J, Jung J, Chang JH, Choi J, Kim H (2021). Exosome-based photoacoustic imaging guided photodynamic and immunotherapy for the treatment of pancreatic cancer. J Control Release.

[127] Xie Y, Bai O, Zhang H, Li W, Xiang J (2010). Tumor necrosis factor gene-engineered J558 tumor cell–released exosomes stimulate tumor antigen P1A-specific CD8+ CTL responses and antitumor immunity. Cancer Biother Radiopharm.

[128] Di Bonito P, Chiozzini C, Arenaccio C, Anticoli S, Manfredi F, Olivetta E, Ferrantelli F, Falcone E, Ruggieri A, Federico M. Antitumor HPV E7-specific CTL activity elicited by in vivo engineered exosomes produced through DNA inoculation. Int J Nanomed. 2017:12:4579–4591. 10.2147/IJN.S131309 PMC5491702 28694699

[129] Hu S, Ma J, Su C, Chen Y, Shu Y, Qi Z, Zhang B, Shi G, Zhang Y, Zhang Y (2021). Engineered exosome-like nanovesicles suppress tumor growth by reprogramming tumor microenvironment and promoting tumor ferroptosis. Acta Biomater.

[130] Huang L, Rong Y, Tang X, Yi K, Qi P, Hou J, Liu W, He Y, Gao X, Yuan C, Wang F (2022). Engineered exosomes as an in situ DC-primed vaccine to boost antitumor immunity in breast cancer. Mol Cancer.

[131] Fan M, Liu H, Yan H, Che R, Jin Y, Yang X, Zhou X, Yang H, Ge K, Liang X-J (2022). A CAR T-inspiring platform based on antibody-engineered exosomes from antigen-feeding dendritic cells for precise solid tumor therapy. Biomaterials.

[132] Zuo B, Zhang Y, Zhao K, Wu L, Qi H, Yang R, Gao X, Geng M, Wu Y, Jing R (2022). Universal immunotherapeutic strategy for hepatocellular carcinoma with exosome vaccines that engage adaptive and innate immune responses. J Hematol Oncol.

[133] Shi X, Cheng Q, Hou T, Han M, Smbatyan G, Lang JE, Epstein AL, Lenz H-J, Zhang Y (2020). Genetically engineered cell-derived nanoparticles for targeted breast cancer immunotherapy. Mol Ther.

[134] Kamerkar S, Leng C, Burenkova O, Jang SC, McCoy C, Zhang K, Dooley K, Kasera S, Zi T, Sisó S (2022). Exosome-mediated genetic reprogramming of tumor-associated macrophages by exoASO-STAT6 leads to potent monotherapy antitumor activity. Science Advances.

[135] Wang J, Wang L, Lin Z, Tao L, Chen M (2014). More efficient induction of antitumor T cell immunity by exosomes from CD40L gene-modified lung tumor cells. Mol Med Rep.

